# Zinner syndrome and infertility─a literature review based on a clinical case

**DOI:** 10.1038/s41443-020-00360-0

**Published:** 2020-11-05

**Authors:** Aybike Hofmann, Franziska Vauth, Wolfgang H. Roesch

**Affiliations:** Department of Pediatric Urology, Clinic St. Hedwig, University Medical Center Regensburg, Regensburg, Germany

**Keywords:** Sexual dysfunction, Reproductive signs and symptoms

## Abstract

Zinner syndrome (ZS) is a rare congenital malformation associated with seminal vesicle cysts, ejaculatory duct obstruction, and ipsilateral renal agenesis. The main treatment focus so far has been on symptomatic patients. Therefore, surgery has been reserved for these patients, and surgical treatment is mainly aimed at pain relief. ZS seems to be frequently associated with infertility, but diagnosing is challenging, particularly during adolescence. This literature review of ZS and infertility is based on the medical report of one adolescent patient.

## Introduction

Zinner syndrome (ZS) is a rare congenital condition characterized by cystic seminal vesicles and ejaculatory duct obstruction (EDO) in association with ipsilateral renal agenesis [1]. Generally, ZS remains asymptomatic until the beginning of sexual activity. EDO leads to seminal fluid accumulation and subsequently to enlarged seminal vesicles [1]. In adolescents, symptoms are generally unspecific and mostly consist of pain, such as dysuria, pollakisuria, perineal pain, epididymitis, and pain after ejaculation [2]. Up to 45% of patients with ZS are affected by infertility [2]. Diagnosing infertility in adolescent patients is challenging. Because surgical treatment is reserved for symptomatic patients, operative management of ZS is primarily concentrated on pain relief and protection of the contralateral ejaculatory duct to preserve fertility. The purpose of the present review based on our patient is to assess the existing data in view of managing ZS and the risk of infertility, especially in adolescent patients.

## Case

**Fig. 1:**
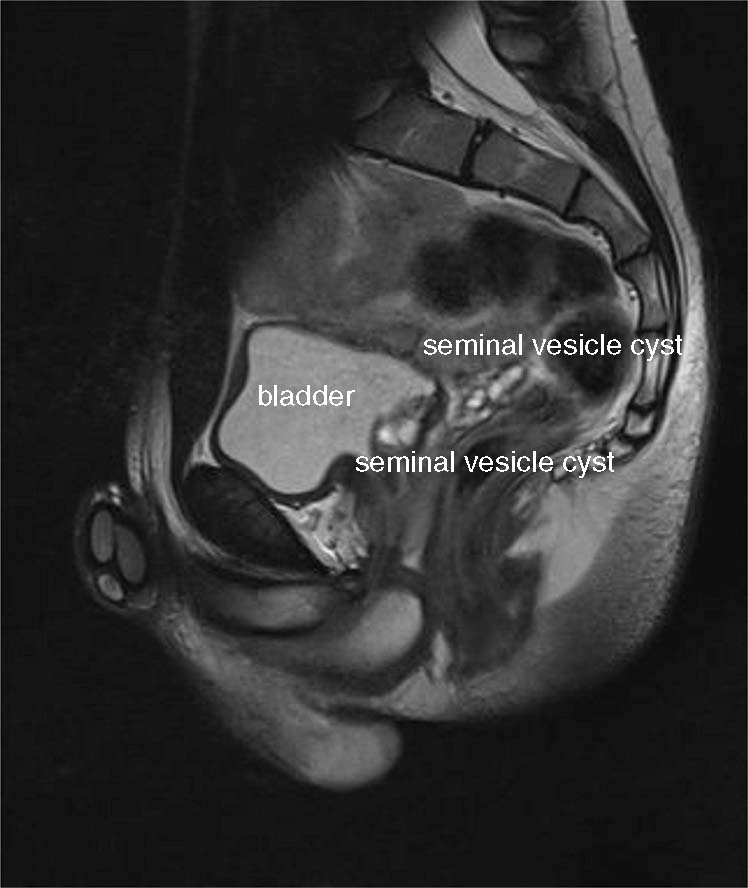
Preoperative MRI of the pelvis - demonstrating enlarged seminal vesicle cysts in the retrovesical space on the left-hand side.

An 18-year-old male patient was referred to our institute because of recurrent macrohematuria. Physical exploration and laboratory evaluation were without pathological findings. Renal and vesical ultrasound showed renal agenesis on the left side and a solid appearing mass in the retrovesical space (3.5 × 1.4 cm). ZS was suspected. Magnetic resonance imaging (MRI) confirmed the presence of enlarged seminal vesicles with intraluminal proteinaceous or hematic content in the retrovesical space on the left-hand side (see Fig. 1). The left kidney was missing, and a residual ureter was visible.

Because of the clinical complaints, the patient underwent transurethral unroofing of the seminal vesicle cysts, which immediately stopped macrohematuria. Because of the predominantly clinical complaints, the ejaculatory duct was not marked with methylene blue. A postoperative transabdominal ultrasound examination did not show any remains of the retrovesical mass. The 24-month follow-up was uneventful. Because ZS has an impact on fertility, the patient was asked to undergo a semen analysis 2 years after surgery. The results of the externally conducted semen analysis are shown in Table 1. Laboratory results (FSH, LH, Prolactin, and free testosterone) were within normal limits. Ultrasound did not show any retrovesical anomalies. The possibility of re-unroofing with methylene blue to investigate patency was discussed. The patient was informed in detail about his ability to father a child. No further investigations were requested by the patient at the time.

**Table 1:** Semen analysis.

Parameter	Value	Reference values
Color	Milky white	
Consistency	Fluidly	
pH	8.1	>7.2
Volume (ml)	2.5	>1.5
Concentration (millions/ml)	1.0	>15
Total spermatozoa (millions)	2.5	>39
Spermatozoa vitality (%)	0	>58
Mobility grade		
Progressive mobility (%)	0	>32
Total mobility	0	>40
Normal appearance (%)	Not measurable	>4

## Embryology, etiology, and pathogenesis

### Zinner syndrome

Seminal vesicle cysts were first described by Smith in 1872. The association of congenital seminal vesicle cysts with unilateral renal agenesis was first reported by Zinner in 1914 and was therefore coined ZS. ZS comprises the triad of cystic seminal vesicles, ipsilateral renal agenesis, and ipsilateral EDO. This association is caused by the maldevelopment of the Wolffian duct between the 4th and 13th week of gestational age [3]. The ureteric bud originates from the proximal portion of the Wolffian duct and fails to join the metanephros due to incomplete migration. As a consequence, the role of the ureteric bud in differentiating the metanephric blastema is disturbed, which leads to ipsilateral renal agenesis and atresia of the ipsilateral ejaculatory duct [1, 4]. The gonad continues to develop, and insufficient drainage of the seminal fluid results in the cystic structure of the seminal vesicle [5].

The incidence of ZS is difficult to determine. More than 200 cases of seminal vesicle cysts associated with ipsilateral renal agenesis have been reported in the literature. When screening 280,000 newborns for renal mass by means of ultrasound scans, Sheih et al. found 13 cases of pelvic dilatations associated with ipsilateral renal agenesis, which implies a frequency of 0.00214% [6]. On the other hand, due to the extensive use of ultrasound scans, the diagnosis of unilateral renal agenesis is becoming more frequent, particularly in fetuses. Prenatal ipsilateral genitourinary malformation should be excluded as it is present in 30–40% of affected fetuses [4].

### EDO and infertility

In ZS, EDO is of congenital origin. EDO can be differentiated between congenital versus acquired, complete versus incomplete, and anatomical versus functional. Functional obstruction is a diagnosis of exclusion and describes the failure of peristalsis of the seminal vesicle [7]. The causes of congenital and acquired cases are stated in Table 2.

**Table 2:** Etiology of EDO.

Congenital	Acquired
Congenital atresia/stenosis	Iatrogenic trauma (i.e., catheterization)
Utricular cysts	Pelvic or bladder surgery
Wolffian duct cysts	Pelvic trauma
Müllerian duct cysts	Genital/urinary tuberculosis

In their evaluation of 87 subfertile patients with EDO, Pryor and Hendry found that the prevalent etiology of EDO was congenital malformation (41%), post-infectious syndromes (22%), trauma (17%), tuberculosis (9%), megavesicles (9%), and neoplastic causes (1%) [7, 8].

Patients with EDO may have azoospermia, severe oligozoospermia, or oligoasthenoterato-zoospermia. In couples examined for infertility, about 15% of the men had azoospermia, which was caused by obstructive azoospermia in 40% of affected men. As a specific type of obstructive azoospermia, EDO is present in 1–5% of infertile men [7].

About 45% of men affected by ZS are infertile [2, 4]. Van den Ouden’s pooled analysis of 52 men found infertility in nine patients, and the fertility status was mentioned in 20 patients. To the best of our knowledge, infertility in association with ZS has been described in six papers, mainly case reports about 14 patients. The largest series has been published by Pace et al. who retrospectively analyzed seven patients affected by ZS and infertility. An overview of the published data is shown in Table 3.

**Table 3:** Literature overview.

Author	Study design	No. of patients	Semen analysis preoperative	Semen analysis postoperative	Treatment
Pereira et al. [4]	Case report	1	OAT	nm	Conservative
Cito et al. [10]	Case report	1	Azoospermia	Azoospermia	RALV
Florim et al. [3]	Case report	1	Normozoospermia	Normal	Conservative
Aghaways and Ahmed [9]	Case report	1	Azoospermia	Normozoospermia	TURED
Pace et al. [18]	Retrospective Study	7	OAT	Improvement but still OAT	TURED
Van den Ouden et al. [2]	Pooled analysis	3	1) OAT 2) Oligozoospermia 3) Azoospermia	nm	nm

Assuming that, in ZS, only one ejaculatory duct is affected, azoospermia should not be expected. However, azoospermia in ZS has been described in several literature reports [9, 10]. The underlying pathogenesis of this aspect is not yet fully understood. One conceivable assumption is that unilateral testicular obstruction may cause antisperm-antibody production, resulting in infertility despite the unobstructed contralateral testis [4]. Cito et al. proposed that—due to long-lasting obstruction—reactive oxygen species may mediate reproductive toxicity in patients with ZS, thus reducing the sperm count by germ cell apoptosis [10]. Another hypothesis is that free semen passage in the normal contralateral duct is blocked because of a congenital defect in the ejaculatory duct area [4].

Nevertheless, further studies are needed to identify the pathomechanism of infertility in patients with ZS. Because of the high rate of infertility associated with ZS, affected patients should be examined regarding their fertility status.

### Clinical presentation

ZS usually remains asymptomatic for a long time. Symptoms tend to only manifest at the start of intensive sexual activity. Literature reports describe a period between the 2nd and 4th decade of life but also an increasingly earlier onset of symptoms in puberty in recent years.

Clinical symptoms are often unspecific and frequently pain-related such as abdominal, pelvic, perineal, or scrotal pain particularly during defecation or ejaculation. Lower urinary tract symptoms such as dysuria, frequency, and urgency have also been reported as well as epididymitis, prostatitis, and recurrent urinary tract infections [1].

Infertility is usually diagnosed in adulthood in the context of the unfulfilled desire to have children. Because ZS seems to have a high impact on fertility, a semen analysis should be conducted when an adolescent patient with ZS reaches adulthood.

### Diagnosis

Diagnostics are usually reserved for patients with symptoms because diagnosing in asymptomatic cases is difficult. In adulthood, transrectal ultrasound (TRUS) is the most commonly used technique for the initial assessment of the seminal vesicle cysts. Because TRUS is not an option for adolescent patients, transabdominal ultrasound scans are used for the first assessment in such cases. Findings include cystic mass in the retrovesical space and the absence of the ipsilateral kidney.

The suspected diagnosis of ZS is usually confirmed by MRI [4]. Seminal vesicle cysts appear as hyperintense on T2-weighted images and as hypointense on T1-weighted images. Seminal vesicle cysts need to be differentiated from other pelvic cystic lesions such as Müllerian duct cysts or utricular cysts. Differentiation is based on the position of the cysts in relation to the bladder neck. Utricular cysts communicate with the urethra, whereas Müllerian duct cysts do not. Müllerian duct cysts have normal seminal vesicles and ejaculatory ducts; anatomically, they are based on the midline. Wolffian ducts cysts, which are present in ZS, are located in the paramedian area [1, 4, 11]. In addition, EDO can be verified by means of MRI.

The standard method for evaluating patients with suspected EDO is open scrotal vasography under fluoroscopic or X-ray control. Avellino et al. showed in their review that TRUS with seminal vesicle aspiration is the most effective method of diagnosis [7]. When comparing TRUS with endorectal MRI, Engin et al. found TRUS to be a reliable method for diagnosing EDO, especially in the case of complete obstruction. On the other hand, MRI is beneficial for examining soft tissue and cystic lesions [12]. In adolescent patients, neither TRUS nor endorectal MRI are feasible methods of diagnosis. Seminal vesicles cysts in combination with ipsilateral renal agenesis detected on conventional abdominal MRI indicate the presence of ipsilateral EDO.

Additional information can be obtained by means of seminal vesicle aspiration. In adult patients, this procedure is conducted under TRUS guidance. In adolescent patients, seminal vesicle aspiration may be carried out intraoperatively before the actual operation. Most patients with EDO show a large number of sperms in the aspirate. The presence of sperm confirms intact spermatogenesis, and sperms can be cryopreserved if necessary [7, 13].

### Management

In ZS, it is sensible to reserve surgical treatment for symptomatic patients, whereas follow-up is acceptable for asymptomatic patients. Surgical treatment options range from transurethral unroofing to open surgery with vesiculectomy with or without vasoligation [1]. Open surgery may be conducted via the transvesical, retropubic, perineal, or transrectal approach [1, 4, 14]. Due to the anatomical position of the seminal vesicles below the urinary bladder, open surgery involves a high risk of injury to related structures such as the bladder neck, external sphincter, and rectum [1, 4, 14].

Minimally invasive surgery has become of growing importance over the past few years. Particularly the robotic approach offers excellent visualization via 3D vision, which leads to lower injury rates because of the possibility of a more detailed dissection of the deep pelvic location [15, 16]. In 2003, Valla et al. performed a laparoscopic excision of progressive seminal cysts in a boy aged 15 months with the aim to preserve fertility. The cystic mass had been diagnosed prenatally and had progressed in size from 12 to 25 mm over a period of 20 months [17]. Unfortunately, no long-term follow-up was reported, particularly regarding the fertility status.

In EDO, standard surgical treatment is the transurethral resection of the ejaculatory duct (TURED). TURED is conducted by cutting the verumontanum at the level of the ejaculatory duct with an electrocautery loop [7]. Methylene blue may be injected into the seminal vesicle to make sure by means of a wash-out that the obstruction has been completely removed [18]. This method is comparable with the method of transurethral unroofing in ZS.

Several publications ranging from case reports to a limited number of larger studies are available on this topic. Larger studies have shown improved semen parameters in 63.0–83.0% of patients with EDO in general, in 90.5% of patients with partial EDO, and in 59.0% of patients with complete EDO. Up to 38% of patients with oligospermia or azoospermia regained normal semen parameters [7, 19–21]. One study showed that surgical treatment did not improve fertility in the presence of congenital abnormalities of the Wolffian duct [7, 8].

## Conclusion

In up to 45% of boys with ZS, infertility due to EDO is a frequent co-existing diagnosis that should be kept in mind when ZS is diagnosed. Because the fertility status is difficult to evaluate in adolescence, these patients should be included in a follow-up program, and a sperm analysis should be conducted when they reach adulthood. Surgical treatment is usually focused on pain relief and should consist of a technique for seminal way disclosure. The high rate of persisting azoospermia after surgical intervention in ZS may be caused by interacting processes affecting contralateral spermiogenesis that are not yet fully understood. Perhaps the currently recommended treatment of ZS that is primarily focused on pain relief may benefit from shifting the focus on the preservation of fertility. Early excision of the affected duct may prevent the negative impact on the contralateral genitourinary tract. Further studies are needed to increase the knowledge on this topic.
