# Correction: Zinner syndrome and infertility—a literature review based on a clinical case

**DOI:** 10.1038/s41443-021-00476-x

**Published:** 2021-10-13

**Authors:** Aybike Hofmann, Franziska Vauth, Wolfgang H. Roesch

**Affiliations:** Department of Pediatric Urology, Clinic St. Hedwig, University Medical Center Regensburg, Regensburg, Germany

**Keywords:** Sexual dysfunction, Reproductive signs and symptoms

Correction to: *International Journal of Impotence Research* 10.1038/s41443-020-00360-0

The article “Zinner syndrome and infertility—a literature review based on a clinical case,” written by Aybike Hofmann, Franziska Vauth, and Wolfgang H. Roesch, was originally published Online First without Open Access. After publication in volume 33, issue 2, page 191–195, the author decided to opt for Open Choice and to make the article an Open Access publication. Therefore, the copyright of the article has been changed to © The Author(s) 2020 and the article is forthwith distributed under the terms of the Creative Commons Attribution 4.0 International License, which permits use, sharing, adaptation, distribution, and reproduction in any medium or format, as long as you give appropriate credit to the original author(s) and the source, provide a link to the Creative Commons license, and indicate if changes were made.

The images or other third-party material in this article are included in the article’s Creative Commons license, unless indicated otherwise in a credit line to the material. If material is not included in the article’s Creative Commons license and your intended use is not permitted by statutory regulation or exceeds the permitted use, you will need to obtain permission directly from the copyright holder.

To view a copy of this license, visit http://creativecommons.org/licenses/by/4.0/.

The original article has been corrected.

